# Correlation between the Serum Pepsinogen I Level and the Symptom Degree in Proton Pump Inhibitor-Users Administered with a Probiotic

**DOI:** 10.3390/ph7070754

**Published:** 2014-06-25

**Authors:** Muneki Igarashi, Jun Nagano, Ayumi Tsuda, Takayoshi Suzuki, Jun Koike, Tetsufumi Uchida, Masashi Matsushima, Tetsuya Mine, Yasuhiro Koga

**Affiliations:** 1 Department of Gastroenterology, Tokai University School of Medicine, Isehara 259-1193, Japan; E-Mails: muneki@tokai-u.jp (M.I); takayoshi@is.icc.u-tokai.ac.jp (T.S.); jkoike@is.icc.u-tokai.ac.jp (J.K.); ucchicchi1025@yahoo.co.jp (T.U); mmatsush@is.icc.u-tokai.ac.jp (M.M.); tetsu-m@is.icc.u-tokai.ac.jp (T.M.); 2 Faculty of Arts and Science, Kyushu University, Kasuga 816-8580, Japan; E-Mail: jun@artsci.kyushu-u.ac.jp; 3 General Internal Medicine, Tokai University School of Medicine, Isehara 259-1193, Japan; E-Mail: ayu0v0ayu@gmail.com; 4 Laboratory for Infectious Diseases, Tokai University School of Medicine, Isehara 259-1193, Japan

**Keywords:** correlation, symptoms, PGI, PPI, LG21

## Abstract

In patients with functional upper gastrointestinal disorders such as gastroesophageal reflux disease and functional dyspepsia, the presence of symptoms is thought to occur in the absence of any organic diseases and the mechanisms behind this remain unclear. We therefore examined the relationship between stomach-related biomarker levels and symptoms. Twenty-four outpatients who had taken proton-pump inhibitors every day were enrolled in this study. The subjects consumed yogurt containing 10^9^ colony-forming units of *Lactobacillus gasseri* OLL2716 (LG21) every day for three months. They underwent four clinical examinations in total. Each examination consisted of answering a questionnaire with a frequency scale for the symptoms of GERD (FSSG), and included measurements of the serum gastrin, ghrelin, and pepsinogens I and II levels. As a result, the FSSG score and the PGI value showed a decrease and an increase, respectively, after LG21 treatment when analyzed without age adjustment. A multiple regression analysis with additional adjustments for gender and age revealed a strong association between the PGI value and the FSSG symptom scores. Therefore either the PGI level itself or the factors regulating the PGI level might be involved in the etiology of these symptoms.

## 1. Introduction

In patients with functional upper gastrointestinal (GI) disorders such as gastroesophageal reflux disease (GERD) and functional dyspepsia (FD), the presence of symptoms is generally thought to occur in the absence of any organic or systematic diseases that are likely to explain the symptoms. In fact, many of the GERD patients complaining of classic symptoms like heartburn have minimal evidence of esophageal mucosal injury although the reflux of acidic contents in the esophagus is suggested to induce the symptoms [1]. Therefore the history of such symptoms plays a crucial role in the diagnosis of GERD. FD is also defined as the presence of symptoms that are thought to originate in the gastro-duodenal region in the absence of any structural lesion there [2]. With regard to the mechanism underlying the symptoms in patients with functional disorders, no sufficient physiological data explaining either the etiology of symptoms or an appropriate target are yet available. As a result, there is a need to develop validated endpoints that may serve as biomarkers for both the diagnosis and treatment of GERD and FD.

Gastrin and pepsinogens are representative biomarkers that influence the gastric physiology and thus reflect the functional state of the gastric mucosa [3]. Gastrin stimulates gastric acid secretion and mucosal cell growth. Pepsinogen (PG) I is secreted in the mucosa of the gastric corpus. PG II is secreted not only in the corpus but also the antrum. As PGs are secreted by chief cells in the gastric mucosa, their serum levels may reflect the mass and/or turnover of those cells in the mucosa. It was reported that measuring these markers in the serum thus allows gastric pathologies such as atrophic gastritis, FD and abnormalities in acid secretion to be detected [4,5].

In the present study, we therefore investigated the relationship between these biomarker levels and the symptoms considered to originate in the esophagus and stomach at several time points in each subject. Moreover, we intended to administer probiotics to the patients to treat their symptoms, because most of them still complained of some symptoms despite taking a proton-pump inhibitor (PPI) every day. We also examined the relationship between the biomarker levels and the symptoms before and after probiotic treatment in each subject.

## 2. Experimental Section

### 2.1. Subjects

A total of 24 outpatients who had taken a PPI every day for more than three months were enrolled in the present study from September 2011 to October 2012. Patients with a history of gastrectomy and those who were younger than 20 years old were excluded. The subjects had been taking a PPI for 7.4 years on average at the time of enrollment. The mean (SD) age of patients was 68.6 (9.7) years old, respectively. The patients consisted of 16 males and eight females. Only one of them was revealed to be serologically positive for anti-*Helicobacter pylori* antibodies. Among the 24 subjects, 13 had GERD, six had chronic gastritis, three had gastric polyp, and two had non-ulcer dyspepsia. The initial diagnosis of GERD was based on the patient’s history or on the responses to a questionnaire, as well as the findings during upper GI endoscopy. Among the 13 GERD patients, seven, three, and three had grade M (minimal change), A, and B/C/D disease, respectively according to the Los Angeles classification. The Ethics Committee of Tokai University Hospital approved the study protocol (11R-065 issued on August 15, 2011), and written consent was obtained from all the participants.

### 2.2. Study Protocol

At first, the subjects consumed 118 g of yogurt containing 10^9^ colony-forming units of *Lactobacillus gasseri* OLL2716 (LG21) every day for a three-month intervention period. The compliance (days of having consumed the yogurt) was evaluated through a diary written by the subject. The subjects underwent four clinical examinations in total: before and after the intervention period, and three and six months after the termination of the intervention. In the morning on the day of the clinical examination, blood was drawn from the subjects after an overnight fast. The subjects were also asked to fill out a questionnaire called the FSSG (frequency scale for the symptoms of GERD) at that time. No explanation was provided but responses were given if questions were asked by the subjects.

### 2.3. The Questionnaire

The FSSG, the questionnaire used in the present study, was originally produced by Kusano *et al.* [6] in order to simply evaluate the symptoms of GERD. This questionnaire comprises 12 questions; seven of which are related to gastroesophageal reflux such as “Do you get heartburn?”, “Do you sometimes subconsciously rub your chest with your hand?”, “Do you get heartburn after meals?”, “Do you have an unusual sensation in your throat?”, “Do some things get stuck when you swallow?”, “Do you get bitter liquid (acid) coming up into your throat?”, and “Do you get heartburn if you bend over?”; five of them were related to dysmotility-like dyspepsia such as “Does your stomach get bloated?”, Does your stomach ever feel heavy after a meal?”, “Do you ever feel sick after meals?”, “Do you feel full while eating meals?”, and “Do you burp a lot?”. Each of these questions was scored to indicate the frequency of symptoms as follows: never = 0; occasionally = 1; sometimes = 2; often = 3; and always = 4.

### 2.4. Laboratory Assays

A serum sample was prepared from whole blood and stored before the analysis at −25 °C for the determination of the serum gastrin, pepsinogen I (PGI), and pepsinogen II (PGII) levels. A plasma sample was also prepared from whole blood to determine the level of des-acyl ghrelin at the same time using a syringe tube containing EDTA-2Na and aprotinin. The gastrin level was assayed with a radioimmunoassay reported elsewhere [7] using an anti-gastrin serum from rabbits immunized with synthetic human gastrin (Imperial Chemical Industries Ltd., Cheshire, UK). The serum selectively recognizes gastrin-17. The PGI and PGII levels were measured by a chemiluminescent enzyme immunoassay using Lumipulse Presto II pepsinogen I (FUJIREBIO Inc, Tokyo, Japan) and Lumipulse Presto II pepsinogen II (FUJIREBIO), respectively [8]. The des-acyl ghrelin level was measured by an ELISA [9] to assess the plasma ghrelin level.

### 2.5. Statistical Analysis

Differences in the distribution of the measurement values between the genders were evaluated using the Wilcoxon rank sum test. Associations between the values and age were assessed based on Spearman’s rank correlation. Differences in the values obtained between before and after LG21 treatment were investigated using the Wilcoxon signed rank sum test. Correlations between the biomarker values and the FSSG scores at the time point of each of the four measurements (1st to 4th) were examined using Spearman’s rank correlation coefficients. Because the patterns in the magnitude and direction of the correlations were similar across the time points, biomarker-FSSG associations were also examined after pooling the data across the four time periods. In order to examine possible independent relationships between the biomarker values and FSSG scores, we used multiple linear regression models that included one of the FSSG symptom scores as a dependent variable and the biomarker values as the independent variables. For this purpose, non-normally distributed variables were log transformed as appropriate. Possible confounding effects were then examined by entering the variables for age and gender as additional independent variables. The reported P values are two-sided, and a *p* value of <0.05 was considered to be statistically significant. The SAS 9.2 for Windows software program was used for the statistical analysis.

## 3. Results

### 3.1. Basal Relationships between the Biomarker Values and FSSG Scores According to Gender and Age

The baseline relationships between the stomach-related biomarker values and FSSG symptom scores according to gender and age are shown in Table 1.

**Table 1:** Baseline relationship between the biomarker values and the FSSG symptom scores according to gender and age ^a)^.

	Gender			Age		
	Male	Female		<70 years	70+ years	
	*N* = 16	*N* = 8		*N* = 13	*N* = 11	
	Median [IQR]	Median [IQR]	Test ^b)^	Median [IQR]	Median [IQR]	Test ^c)^
**Biomarker values**						
Gastrin pg/mL	235.0 [120.0–470.0]	275.0 [180.0–660.0]	NS	200.0 [110.0–450.0]	430.0 [230.0–840.0]	*p* < 0.05
Ghrelin pg/mL	138.5 [77.0–188.0]	375.0 [170.5–599.0]	NS	160.0 [79.0–195.0]	195.0 [73.0–438.0]	NS
PGI ng/mL	126.0 [73.6–253.5]	144.0 [71.4–191.0]	NS	116.0 [74.7–179.0]	136.0 [68.0–266.0]	NS
PGI/II ratio	5.70 [5.15–7.25]	6.80 [5.95–8.50]	NS	6.30 [5.60–8.20]	5.60 [5.20–7.20]	NS
**FSSG scores**						
Total symptoms	6.5 [3.0–15.5]	12.5 [5.5–16.5]	NS	12.0 [8.0–19.0]	4.0 [2.0–14.0]	*p* < 0.05
Reflux symptoms	3.0 [1.0–8.5]	8.5 [3.0–11.0]	NS	8.0 [4.0–11.0]	2.0 [0.0–7.0]	*p* < 0.05
Dysmotility symptoms	3.5 [1.0–7.5]	3.5 [2.0–6.5]	NS	5.0 [3.0–8.0]	3.0 [2.0–5.0]	*p* < 0.05

^a)^ All the data were from the 1st examination. ^b)^ Test for difference based on the Wilcoxon rank sum test. ^c)^ Test for association based on Spearman’s rank correlation. IQR: interquartile range. NS: Not significant.

No significant differences were observed between the male and female subjects with respect to the biomarkers or symptoms. On the other hand, an age greater than 70 years was found to be associated with a higher level of gastrin, although no relationships were found for the ghrelin and PGI levels or PGI/II ratio. Moreover, an older age was found to be associated with reduced scores for all symptoms.

### 3.2. Effects of the LG21 Treatment on the Biomarker Values and the FSSG Score

**Figure 1:**
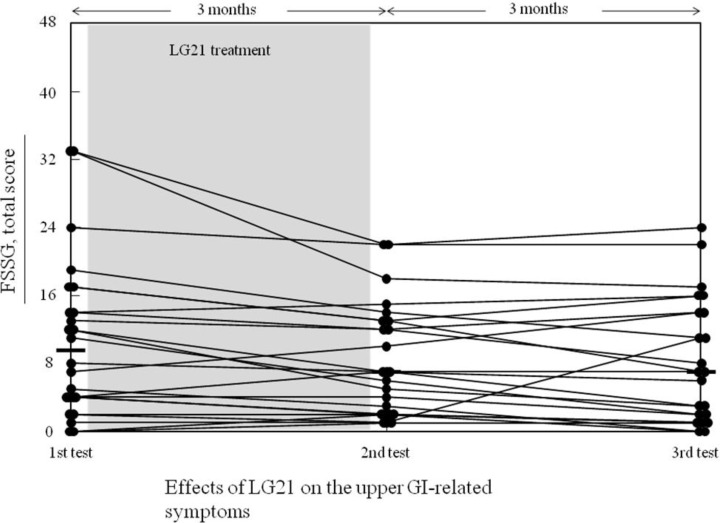
Effects of LG21 on the upper GI-related symptoms.

The 24 subjects taking PPI in everyday clinical practice were further treated with a probiotic strain, LG21, in yogurt for 3 months, and their stomach-related biomarkers and upper GI tract-related symptoms were examined before (1st examination) and after (2nd examination) the intervention (Table 2). All the subjects completed the LG21 treatment, and the compliance was more than 90% for all of them. While the PGI level tended to increase following the LG21 treatment when compared by both the median and mean values, no statistically significant difference was found between these examinations. On the other hand, the FSSG total score showed a considerable decrease after LG21 treatment when analyzed without age adjustment (Figure 1, Table 2). Such tendency of improvement in the score was still found 3 months after the end of the intervention, and the median of total, reflux, and dysmotility scores were 7.0, 2.5, and 3.0, respectively. Although the median of the dysmotility score was higher after the LG21 treatment than before the treatment (Table 2), the individual score decreased in 12 subjects (50%) while unchanged in 6 (25%) and increased only in 6 (25%). After all, a significant improvement was not observed any more in 3 or 6 months follow-up (data not shown).

At first, twenty-four subjects consumed yogurt containing 10^9^ CFU of LG21 everyday for three months. The subjects underwent clinical examinations before and after LG21 treatment, and three months after the termination of the intervention. The dots and lines represent the FSSG total score of each subject.

**Table 2:** Effect of the LG21 treatment on the biomarker values and the FSSG scores.

	1st Examination (Before LG21 Treatment) *N* = 24	2nd Examination (After LG21 Treatment) *N* = 24	Test ^a)^
**Biomarkers**			
Gastrin, pg/mL	250.0 [140.0–485.0] ^b)^, 388.6 (365.7)	250.0 [125.0–585.0] ^b)^, 359.6 (273.4)	*p* = 0.67
Ghrelin, pg/mL	169.5 [77.0–375.0] ^b)^, 268.3 (271.8)	172.0 [74.0–260.0] ^b)^, 233.2 (212.0)	*p* = 0.25
PGI, ng/mL	126.0 [71.4–208.5] ^b)^, 145.9 (90.0)	139.5 [75.0–201.5] ^b)^, 155.6 (105.5)	*p* = 0.27
PG-I/II, ratio	6.4 (1.6) ^c)^	6.2 (1.7) ^c)^	*p* = 0.21
**FSSG scores**			
Total, 0–48	9.5 [4.0–15.5], 10.8 (0.5) ^c)^	7.0 [2.0–13.0], 8.4 (6.6) ^c)^	*p* = 0.005
Reflux, 0–28	4.0 [1.0–10.5], 6.2 (6.2) ^c)^	3.0 [1.0–8.0], 4.8 (4.7) ^c)^	*p* = 0.008
Dysmotility, 0–20	3.5 [2.0–7.5], 4.6 (3.8) ^c)^	4.0 [2.0–5.5], 3.6 (2.5) ^c)^	*p* = 0.021

^a)^ Test for difference based on the Wilcoxon signed rank sum test. ^b)^ Median [interquartile range]. ^c)^ Mean (standard deviation).

### 3.3. Relationships between the Symptoms and the Biomarkers

We then statistically analyzed the correlations between the stomach-related biomarkers and FSSG scores in the four independent examinations using Spearman’s correlation test. As a result, weak but not significant inverse correlations were observed between the serum gastrin level and the reflux symptom score across the four measurements. On the other hand, moderate correlations were found between the PGI level and the dysmotility symptom score in both the 1st and 2nd examinations (Table 3). However, no significant correlations were noted between the symptom scores and the ghrelin level or PGI/II ratio (data not shown). When all data were pooled across the four time measurements (Table 4), the correlations between the symptom scores and the gastrin and PGI levels were statistically significant. No significant correlations were observed for the ghrelin level or PGI/II ratio. We then examined possible independent associations between the gastrin and PGI levels and the symptom scores using multiple linear regression models (Table 5). In this analysis, we log transformed all non–normally distributed variables for biomarkers and symptoms, as the residual analyses indicated that transformation improved the fitness of the models. With regard to the total and dysmotility symptoms, both the gastrin and PGI levels showed a significant reverse correlation with these symptoms in the models including only the biomarker values. However, an additional adjustment for gender and age eliminated the association with the gastrin level, while the associations with the PGI level and age remained with high statistical significance (*p* < 0.001). As for the reflux symptoms, the model including gender and age as well as two biomarkers showed that only the PGI level (*p* = 0.015) and age (*p* < 0.001) exhibited significant inverse correlations with the symptom scores.

**Table 3:** Associations between upper GI symptoms and biomarkers at each time point; sex and age adjusted.

Exam. No.^a)^	Symptoms	Biomarker	*t*	*p*	Adjusted R^2^
1st	Reflux	Gastrin	−0.70	0.49	0.29
		PGI	−1.48	0.15	
	Dysmotility	Gastrin	0.53	0.61	0.30
		PGI	−2.40	**0.027**	
2nd	Reflux	Gastrin	−1.14	0.27	0.26
		PGI	−0.85	0.41	
	Dysmotility	Gastrin	−0.06	0.95	0.32
		PGI	−2.45	**0.024**	
3rd	Reflux	Gastrin	−1.49	0.15	0.30
		PGI	−0.77	0.45	
	Dysmotility	Gastrin	−0.14	0.89	0.21
		PGI	−0.72	0.48	
4th	Reflux	Gastrin	−1.02	0.32	0.13
		PGI	−1.66	0.12	
	Dysmotility	Gastrin	0.45	0.66	−0.13
		PGI	−1.26	0.23	

a) 1st, Pre-probiotic; 2nd, Post-probiotic; 3rd, 3 months follow-up; 4th, 6 months follow-up.

**Table 4:** Correlation between the stomach-related biomarkers and the FSSG scores.

Biomarkers	N	FSSG symptoms score		
		Total	Reflux	Dysmotility
Gastrin	91	−0.32 ^a)^**	−0.36 ***	−0.22 *
Ghrelin	93	−0.01	0.01	−0.06
PGI	93	−0.35 ***	−0.26 *	−0.30 **
PGI/II	93	0.04	0.16	−0.08

^a)^ Spearman’s rank correlation coefficients; * *p* < 0.05, ** *p* < 0.01, *** *p* < 0.001.

**Table 5:** Associations between upper GI symptoms and biomarkers: Multiple regression analysis.

Upper GI symptoms	Model			
	Biomarkers only		Sex and age adjusted	
	*t*	*p*	*t*	*p*
*Total symptoms*				
Gastrin	−3.03	0.003	−1.06	0.29
PGI	−2.85	0.005	−3.74	<0.001
Female sex			1.31	0.19
Age			−3.85	<0.001
	R^2^ = 0.168		R^2^ = 0.331	
	Adjusted R^2^ = 0.150		Adjusted R^2^ = 0.300	
*Reflux symptoms*				
Gastrin	−3.38	0.001	−1.27	0.21
PGI	−1.60	0.11	−2.47	0.015
Female sex			1.45	0.15
Age			−4.23	<0.001
	R^2^ = 0.139		R^2^ = 0.334	
	Adjusted R^2^ = 0.120		Adjusted R^2^ = 0.304	
*Dysmotility symptoms*				
Gastrin	−2.02	0.046	−0.14	0.89
PGI	−2.72	0.008	−3.44	<0.001
Female sex			0.13	0.89
Age			−3.50	<0.001
	R^2^ = 0.118		R^2^ = 0.237	
	Adjusted R^2^ = 0.099		Adjusted R^2^ = 0.202	

The symptom scores and the values for gastrin and PGI were log-transformed.

## 4. Discussion

In the present study including PPI-users as the subjects, the administration of a probiotic strain, LG21, was strongly suggested to improve the upper GI tract-related symptoms that had persisted in spite of the use of a PPI every day, whereas interpretation of the result takes caution because the present study lacked a control group. In a previous study on *H. pylori*-infected individuals, on the other hand, the efficiency of LG21 for the treatment of stomach-related symptoms was demonstrated by a double-blinded randomized controlled trial [10]. In this study, LG21 was revealed to be effective for suppressing the symptoms of postprandial distress syndrome such as fullness and bloating. While it still remains unclear how LG21 improved these symptoms, a mechanism other than gastric acid suppression is suggested because LG21 could still alleviate the symptoms in the patients who had been treated by a PPI in everyday clinical practice. The stomach-related biomarker values showed some change in association with the improvement of the symptoms by LG21 treatment. Therefore we investigated the relationship between the symptoms and these gastric biomarkers to elucidate the reasons for such improvement.

In the analysis using both Spearman’s correlation and the multiple regression tests, a significant correlation was found between the PGI level and the symptoms, especially the dysmotility symptom. PGI is predominantly secreted into the gastric lumen by chief cells and is detectable in the serum. PGs are then degraded auto-catalytically in the acidic environment to form a number of active pepsins that in turn initiate the digestion of ingested proteins in the stomach [11]. Therefore if the PGI level was directly involved in the occurrence of the symptoms, an increase in the PGI/pepsin level and thus the acceleration of the digestion of food proteins in the stomach might be one of the reasons to explain the association between the higher PGI level and the amelioration of symptoms in our patients, especially in the case of dysmotility symptoms.

Another possible reason might be that a common factor is involved in both the regulation of the serum PGI level and the degree of the symptoms. Long-term therapy with PPI is known to be associated with increased levels of PGI, an effect probably mediated by the associated hyper-gastrinemia [12]. In the present study, all of the subjects had been treated with a PPI for several years or more and many of them had already exhibited a high level of both gastrin and PGI at the start of the LG21 treatment. However, LG21 treatment exerted no change on the gastrin level whereas this treatment tended to further increase the PGI level. Therefore, it is unlikely that gastrin is a common factor that improves the symptoms and increases the level of PGI. Uchida *et al.* [13] reported that yogurt containing LG21 significantly increased the prostaglandin E_2_ generation in the gastric mucosa in rats. Prostaglandins, such as prostaglandin E_2_ and prostaglandin I_2_, are known to exert gastro-protective effect by increasing the gastric mucosal blood flow or bicarbonate secretion. Moreover, prostaglandin E_2_ is reported to stimulate chief cells to secrete PGI via the cAMP pathway [14]. Taken together, these data suggest the possibility that LG21 induced the generation of prostaglandin E_2_, which then improved the symptoms via its gastro-protective effects and also increased the PGI level in the stomach in the present study.

Of note was that a strong reverse correlation was found between age and the symptom scores in the present study. The risk of developing gastric atrophy is one of the pathophysiological features of PPI-users [15]. As all the subjects were chronic PPI-users, the older subjects tended to have used PPI longer and thus had a higher risk of advanced atrophy. Such atrophic gastric mucosa will secrete less amount of gastric acid, and may be insensitive to recognizing the disorders causing stomach-related symptoms. The development of dementia with aging might also have caused a loss in the sensation of the symptoms in the older subjects.

## 5. Conclusions

In conclusion, the present study suggested a significant association to exist between the serum PGI level and the symptoms observed in PPI-users. Further studies with a refined design and examining a larger number of subjects will thus be needed to confirm our findings. Since there are currently no data available regarding treatment approaches for these functional disorders, agents which tend to increase the serum PGI level such as LG21 may also be candidates for the treatment of these patients.
